# Exploring the Thickness-Dependence of the Properties of Layered Gallium Sulfide

**DOI:** 10.3389/fchem.2021.781467

**Published:** 2021-11-19

**Authors:** Yael Gutiérrez, Maria M. Giangregorio, Stefano Dicorato, Fabio Palumbo, Maria Losurdo

**Affiliations:** Institute of Nanotechnology, CNR-NANOTEC, c/o Dipartimento di Chimica, Università di Bari, Bari, Italy

**Keywords:** layered GaS, chalcogenides, work function, optical properties, photoresistivity

## Abstract

Group III layered monochalcogenide gallium sulfide, GaS, is one of the latest additions to the two-dimensional (2D) materials family, and of particular interest for visible-UV optoelectronic applications due to its wide bandgap energy in the range 2.35–3.05 eV going from bulk to monolayer. Interestingly, when going to the few-layer regime, changes in the electronic structure occur, resulting in a change in the properties of the material. Therefore, a systematic study on the thickness dependence of the different properties of GaS is needed. Here, we analyze mechanically exfoliated GaS layers transferred to glass substrates. Specifically, we report the dependence of the Raman spectra, photoluminescence, optical transmittance, resistivity, and work function on the thickness of GaS. Those findings can be used as guidance in designing devices based on GaS.

## Introduction

Low-dimensional layered semiconductors are receiving increasing interest due to the possibility to tailor their light–matter interaction by varying their properties with the number of layers, especially in the few-layer regime. In this context, semiconducting gallium monochalcogenides such as gallium sulfide, selenide, telluride, GaX (X = S, Se, Te) are one of the latest additions to the two-dimensional (2D) materials family, and of particular interest for visible-UV optoelectronic applications due to their wide energy bandgap (Lu et al., 2020). Specifically, gallium monosulfide, GaS, in the bulk form, has an indirect bandgap of 2.35 eV corresponding to the electronic transition from **Γ**→M points in the band structure (Chen et al., 2015), whereas the bandgap of GaS monolayer has been calculated to be above 3 eV, with different values reported in the range 3.1–3.3 eV (Zhuang and Hennig, 2013; Jung et al., 2015). A direct bandgap (**Γ**→**Γ** transition) in the range 2.8–3.0 eV has also been reported for bulk GaS (Kepinska et al., 2001; Ho and Lin, 2006) and at 3.88 eV for the monolayer (Jung et al., 2015). Because of those bandgap values, GaS has potential to exhibit photoluminescence (PL) in the green-blue spectral region and to be exploited as a UV photodetector (Chen et al., 2019; Lu et al., 2020).

**FIGURE 1:**
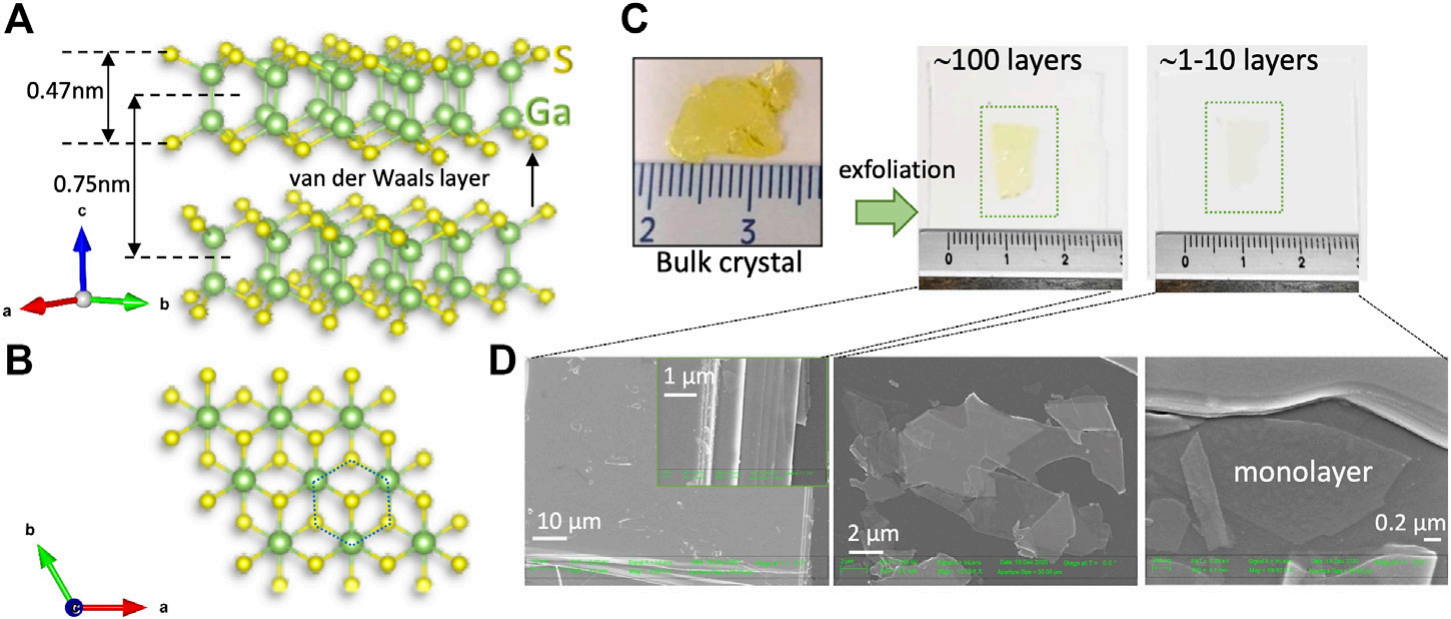
**(A)** Side and **(B)** top view of the crystalline structure of 2H-GaS. **(C)** Images of the yellow bulk GaS crystal and of the mechanical exfoliated layers on glass substrate, showing the reduction of the yellow color by reducing the number of layers. **(D)** SEM images of the exfoliated GaS showing its layered structure down to monolayer. The inset in **(D)** is for the multilayer border where the piling up of the various layers can be clearly seen and layers can be counted.

GaS crystallizes in a highly anisotropic layered structure of increasing interest due to its non-toxicity, high chemical and thermal stability, and resistance to oxidation. Specifically, the basal surface of the layered structure, shown in Figure 1, is extremely inert to chemisorption of contaminants as the sticking coefficient for contaminants on GaS has been reported to be undetectably small (Williams and McEvoy, 1972); consequently, contaminants are only loosely bound on GaS basal surface, and, hence, can easily be removed by heating in vacuum or exposure to an electron beam (Williams et al., 1972).

Within the GaS layer, there is strong covalent binding, with two covalently bonded gallium, Ga, atoms between two layers of sulfur, S, atoms (see Figure 1A). Conversely, the inter-layers binding is of the Van der Waals type, classifying GaS as a 2D Van der Waals semiconductor. The unit cell of GaS is hexagonal, with the 2H-phase, β-GaS, crystal structure being the most energetically favorable polytype with lattice constants *a* = *b* = 3.587 Å, *c* = 15.492 Å. The interlayer separation of ∼0.75 nm is shown in Figure 1. Interestingly, the formation energy of a monolayer of GaS has been calculated to be 0.06 eV/atom, which is even lower than that of MoS_2_ (0.08 eV/atom) (Zhuang and Hennig, 2013), indicating that monolayer GaS can be obtained by mechanical exfoliation, as shown in Figures 1C,D.

In this work, we report a survey on the dependence of the main structural, optical, and electrical properties of mechanically exfoliated GaS on thickness, from bulk down to monolayer. This knowledge is relevant for designing devices and applications exploiting different thicknesses of GaS.

As Raman spectroscopy is the prime non-destructive characterization technique for layered materials, we report the Raman and PL data acquired at the same spot as a function of thickness from bulk GaS to monolayer. Furthermore, one of the most critical parameters in the design of novel electronic devices based on semiconducting layered materials is the work function (WF). This parameter is relevant for designing and understanding the band alignment at metal–semiconductor interfaces and in semiconducting heterostructures for photodetectors or phototransistors (Liu et al., 2021). For layered materials at the ultrathin regime, the WF is expected to critically depend on the number of layers of the material. For instance, it has been demonstrated that, for the prototypical transition metal dichalcogenide MoS_2_, the WF increases monotonically with the increase in the number of layers (Li et al., 2013; Choi et al., 2014). To the best of our knowledge, such study has not yet been performed on GaS; hence, we report the dependence of the GaS WF as a function of the GaS thickness as obtained by Kelvin probe force microscopy (KPFM). Moreover, x-ray photoelectron spectroscopy analysis of the valence band (VB) has been used to analyze the position of the valence band maximum (VBM) with respect to the Fermi level (E_F_) and corroborate the KPFM data for profiling bands as a function of thickness. To further guide the design of optoelectronic devices, we provide values of the resistivity and transmittance of GaS as a function of thickness in dark and under visible and UV illumination.

## Materials and Methods

### Sample Fabrication

Few-layer GaS samples were obtained by mechanical exfoliation from commercially available bulk crystals purchased from 2D Semiconductors and HQ Graphene and transferred to glass and (285 nm) SiO_2_/Si substrate by the thermal tape method. Several exfoliations were executed in order to obtain samples of decreasing thickness as inferred by the disappearing of the yellow color characteristics of GaS bulk crystal, as shown in Figure 1. Glass substrates were cleaned by diluted H_2_O:H_2_O_2_ for 1 h at room temperature, followed by a water rinse to obtain an -OH terminated surface to improve adhesion with GaS.

### Number of Layers and Thickness by Scanning Electron Microscopy

Scanning electron microscopy (SEM) was carried out for the morphological characterization of the samples with a Zeiss Supra 40 FEG SEM equipped with a Gemini field emission gun. Analyses were carried out at an extraction voltage of 3 kV and a 30-µm aperture.

### Structure and Photoluminescence by Raman Spectroscopy

Raman spectroscopy (LabRam Horiba) was performed using a ×100 microscope objective (NA = 0.9) and exciting wavelengths of 633 and 473 nm. For the Raman measurements, performed with an excitation wavelength of 633 nm, the exfoliated flakes were deposited on glass substrates to avoid misinterpreting and overlapping of one of the GaS Raman modes with that at 303 cm^−1^ of conventionally used SiO_2_/Si substrates. The 473-nm laser was used to excite the GaS above the bandgap and also acquire PL spectra.

### Work Function and Morphology by Kelvin Probe and Atomic Force Microscopy

The WF of GaS flakes with different thicknesses was measured by Kelvin probe electrical force microscopy (KPFM) using the Autoprobe CP (Thermomicroscope) through the measurement of the local variation of the surface potential (SP). The sample topography and SP were recorded in a single-pass mode using gold-coated Si tips (their frequency is ∼80 Hz) in non-contact mode. The oscillating potential, *V*_ac_, applied to the tip is 5 V at a frequency *ω* of 13 kHz. The samples were electrically connected to the ground of the microscope (the sample stage).

For the Kelvin probe force microscopy experiments, the flakes were deposited on a reference Au/Si substrate, as the WF of gold at 4.75 eV (as measured by us on the same equipment and corroborated by x-ray photoelectron spectroscopy measurements) was used as reference. All measurements were collected in air at room temperature.

### Valence Band Analysis by X-Ray Photoelectron Spectroscopy

For profiling the bands’ energy levels, we determined the position of the VBM with respect to the Fermi level by x-ray photoelectron spectroscopy (XPS) using a Scanning XPS Microprobe (PHI 5000 Versa Probe II, Physical Electronics) equipped with a monochromatic Al Kα x-ray source (1,486.6 eV), with a spot size of 200 µm. Survey (0–1,200 eV) and high-resolution spectra (C 1s, O 1s, S2p, S2s, Ga2p3, Ga3d, and valence band region) were recorded in FAT mode at a pass energy of 117.40 and 29.35 eV, respectively. Spectra were acquired at a take-off angle of 45° with respect to the sample surface. Surface charging was compensated using a dual beam charge neutralization system, and the hydrocarbon component of C1s spectrum was used as internal standard for charging correction, and it was fixed at 285 eV.

### Electrical Resistivity and Optical Transmittance Measurements

Electrical current–voltage, *I–V*, measurements were performed by the Keithley617 Programmable Electrometer. The voltage source has been used in conjunction with the electrometer section, to apply to the samples voltages from −2 to +2 V, where GaS has ohmic behavior. Contacts were made using silver. Current was measured in the dark whereas photoresponse was investigated in the visible range under a 100 mW·cm^−2^ AM1.5 spectrum lamp and in the UV range using a 405-nm laser of 250 mW cm^−2^ as source.

UV–Vis transmittance spectra were measured on the same glass samples with a Perkin Elmer Lambda 900 spectrometer.

## Results

### Colorimetry for Thickness Determination

**FIGURE 2:**
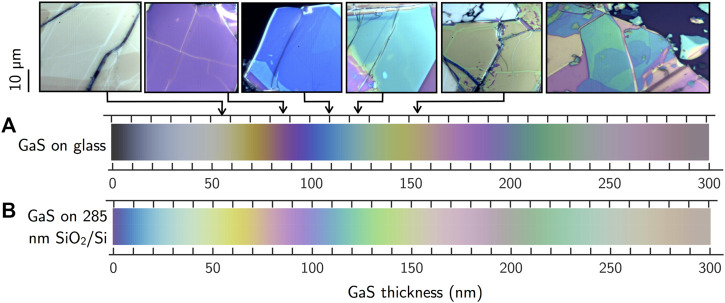
Color evolution of GaS flakes as a function of their thickness on **(A)** glass and on **(B)** 285-nm SiO_2_/Si substrates as determined according to the free-available code developed (Gutiérrez, 2021). The last image on the right is an example of an optical micrograph of a non-homogeneous exfoliated flake, with the different colors corresponding to different number of layers.

Although mechanical exfoliation has become a widely used technique to achieve 2D layers, one of its main drawbacks is the difficulty in obtaining large area samples with homogeneous number of layers (as it can also be inferred by Figure 1D), and it generally results in randomly distributed flakes of different thickness. Consequently, there is a need for non-destructive, reliable, effective, and fast methods for inferring thickness. Interestingly, because of the contrast in the optical properties between GaS flake and its substrate, a full gamut of colors allows one to identify the thickness of mechanically exfoliated GaS transferred onto substrates, as shown in Figure 2. Optical microscopy methods relying on colorimetry can provide an effective solution to this problem. By calculating the reflectance of a system consisting of a GaS layer of variable thickness on an infinite substrate using a Fresnel laws-based model, and then its conversion to color coordinates using color matching functions, it is possible to predict the apparent color of a GaS flake of a specific thickness on a given substrate. Following this procedure, we have developed a methodology (Gutierrez et al., 2021) and a code (Gutiérrez, 2021) that generates color rulers for the quick assessment of the thickness of GaS flakes on various substrates. As an example, Figure 2 shows the color evolution of GaS as a function of thickness on glass and on 285-nm SiO_2_/Si substrates as seen under an optical microscope when running a Raman measurement. By comparing the color of the flakes appearing under the microscope of the Raman system with those color rulers, it is possible to infer the thickness of the GaS flakes.

### Raman and Photoluminescence Spectra

**FIGURE 3:**
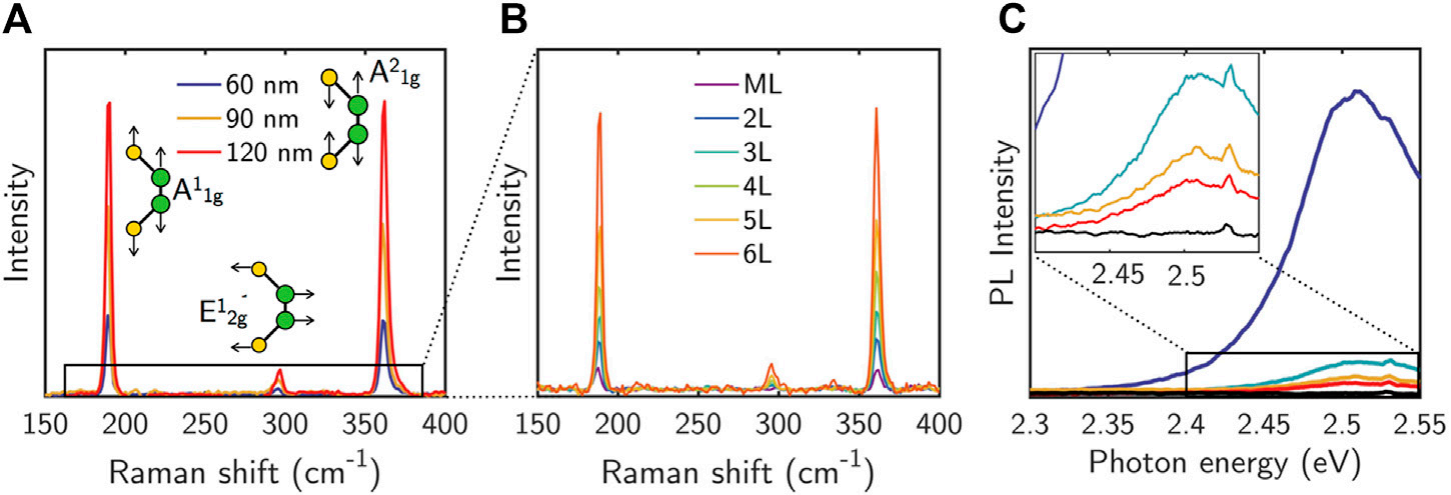
Typical Raman spectra of **(A)** thick GaS and **(B)** few-layer GaS raging from the monolayer (ML) to six layers transferred onto glass substrates (acquired using a 633-nm laser source). **(C)** PL spectra of same samples with different thickness acquired irradiating the sample with a 473-nm laser. The blue intense spectrum is for a >300-nm-thick sample; the black line is for the 1–6 L samples. Intermediate states are for layers with a thickness of ≈300 nm.

Figure 3 shows the thickness dependence of the Raman spectra of few-layer GaS samples. The Raman spectrum of bulk β-GaS (space group P6_3_/mmc and point group D^4^_6h_), as well as of thick layers, is characterized by six modes at 22.8 cm^−1^ ( $E_{2g}^{2}$ ), 74.7 cm^−1^ ( $E_{1g}^{1}$ ), 189 cm^−1^ ( $A_{1g}^{1}$ ), 291.8 cm^−1^ ( $E_{1g}^{2}$ ), 295.8 cm^−1^ ( $E_{2g}^{1}$ ), and 360.9 cm^−1^ ( $A_{1g}^{2}$ ). The most intense and investigated peaks are the $A_{1g}^{1}$ , $A_{1g}^{2}$ , and $E_{2g}^{1}$ (the latter often including the contribution of the nearby $E_{1g}^{2}$ ), whose vibrational modes are sketched in Figure 3A. Conversely, GaS monolayer (space group P-6m2 and point group D^1^_3h_) shows the $E_{1g}^{2}$ barely distinguishable as shown in Figure 3B. For a more accurate analysis, line-shape analysis for each Raman mode was performed by using one Lorentzian component. Central Raman shifts (ω) and the full width at half maximum FWHM (Γ) for each of the spectra recorded are shown in Table 1. No significant variation in the peaks position can be observed for all modes; similarly, the FWHM variation from thick layers to monolayer is within 1 cm^−1^. This negligible dependence of Raman modes on thickness is mainly due to weak inter-layer interactions, and it is consistent with a previous work where it is reported a red-shift in the $A_{1g}^{1}$ mode of only 1.4 cm^−1^ when going from the monolayer (187.6 ± 0.3 cm^−1^) to a 38-nm-thick layer (189.0 ± 0.1 cm^−1^), whereas a constant position within the uncertainty was reported for $A_{1g}^{2}$ (Alencar et al., 2020). Similarly, the broadening of all Raman modes is within 1 cm^−1^ going from monolayer to bulk. It is worth mentioning that for high-crystalline quality GaS, the 1:1 ratio between $A_{1g}^{1}$ and $A_{1g}^{2}$ is preserved in the range of thickness from bulk to monolayer, indicating that the stacking order of the layers is preserved during exfoliation.

**TABLE 1:** Raman shifts (*ω*) and FWHM (Γ) for the three modes $A_{1g}^{1}$ , $E_{2g}^{1}$ , and $A_{1g}^{2}$ measured for GaS layers with thickness ranging from monolayer to 120 nm. The 633-nm laser excitation was used.

	$A_{1g}^{1}$		$E_{2g}^{1}$		$A_{1g}^{2}$	
	Γ (cm^−1^)	ω (cm^−1^)	Γ (cm^−1^)	ω (cm^−1^)	Γ (cm^−1^)	ω (cm^−1^)
ML	3.4 ± 0.8	187.4 ± 0.8	−	−	3.1 ± 0.8	361.6 ± 0.8
2L	4.0 ± 0.5	188.4 ± 0.5	5.1 ± 0.8	296.5 ± 0.8	3.5 ± 0.5	361.5 ± 0.5
3L	3.2 ± 0.5	188.5 ± 0.5	4.6 ± 0.8	295.9 ± 0.8	4.2 ± 0.5	361.6 ± 0.5
4L	3.5 ± 0.5	188.4 ± 0.5	3.8 ± 0.5	296.1 ± 0.5	4.1 ± 0.5	361.4 ± 0.5
5L	2.7 ± 0.5	188.6 ± 0.5	3.9 ± 0.5	295.9 ± 0.5	3.7 ± 0.5	361.3 ± 0.5
6L	2.8 ± 0.5	188.6 ± 0.5	4.1 ± 0.5	295.3 ± 0.5	3.9 ± 0.5	361.1 ± 0.5
60 nm	2.9 ± 0.5	188.6 ± 0.5	3.6 ± 0.5	295.1 ± 0.5	4.1 ± 0.5	360.6 ± 0.5
90 nm	2.8 ± 0.5	188.8 ± 0.5	4.6 ± 0.5	2.95.1 ± 0.5	4.0 ± 0.5	361.3 ± 0.5
120 nm	2.8 ± 0.5	188.7 ± 0.5	4.4 ± 0.5	295.4 ± 0.5	4.0 ± 0.5	360.9 ± 0.5

Literature has given little attention to the PL of GaS. Figure 3C shows the PL spectra for samples of different thickness using a continuous-wave excitation from a 473-nm laser source. Noteworthy, the monolayer GaS as well as all the layered samples up to approximately 300 nm do not show any PL. A sharp PL peak at approximately 2.5 eV starts to be seen for thicknesses above 300 nm and increases with the increase in thickness, as shown in Figure 3C. This can be explained considering two main factors: 1) GaS is an indirect bandgap semiconductor requiring both photons and phonons for radiative recombination and defect-assisted recombination plays an important role, as due to the requirement of phonon momentum conservation, the radiative recombination on the indirect transition will be inefficient and sensitive to traps (Leonhardt et al., 2020). For monolayer and few layers, if the trap density is mainly localized at the substrate/GaS interface, traps result in the observed quenching of PL for the few-layer regime, as the substrate-interaction traps act as recombination centers in the bandgap. This is supported by literature (Shin et al., 2016), reporting that the surface roughness of the underlying substrate can result in inhomogeneous strain that leads to bandgap modifications in thin transition metal dichalcogenides causing the appearance of hole traps. The origin of these traps, however, is still under investigation. 2) By increasing the thickness, both the direct and indirect bandgaps are affected by the interlayer interactions along the *c*-axis and the appearance of new radiative recombination paths. This condition leads to the appearance of a distinct PL peak.

### Optical and Electrical Properties

**FIGURE 4:**
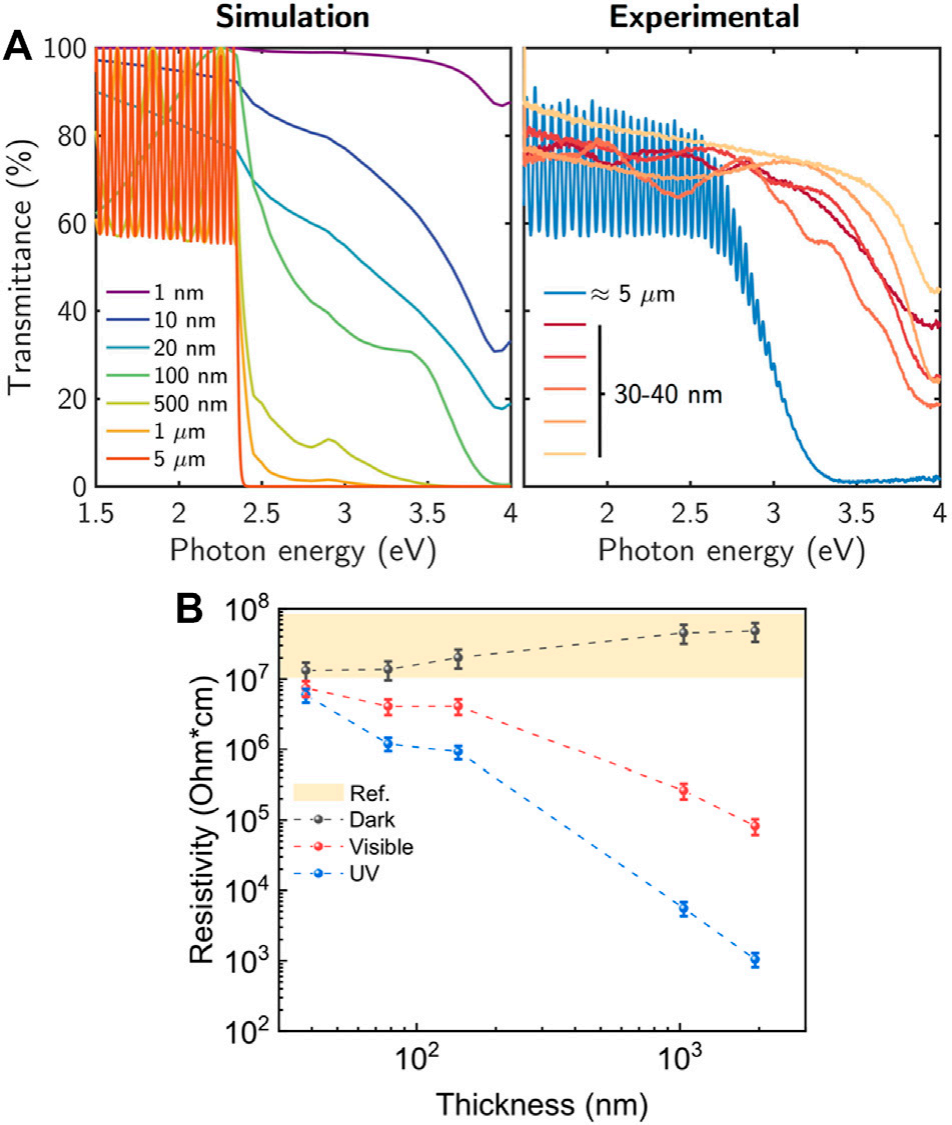
**(A)** Calculated and measured transmittance as a function of the GaS thickness. **(B)** Thickness dependence of the resistivity of GaS layers under dark, visible-, and UV-light irradiation. For comparison, the yellow shadowed area indicates the range of the dark resistivity values reported in literature for GaS (Manfredotti et al., 1976; Mancini et al., 1983; Micocci et al., 1990; Szałajko and Nowak, 2007).

Figure 4 shows typical optical transmittance spectra measured on 30–40 nm and ≈5-µm-thick GaS. Simulated optical transmittance spectra are also shown as reference for different GaS thicknesses from 1 Ml to 5 μm. The simulations were performed using the Transfer Matrix Method (Born et al., 1999) with the assumption of a flat interface multilayer GaS/glass system. The optical constants of GaS used in the simulations were experimentally measured by spectroscopic ellipsometry on a bulk crystal *c*-axis oriented. Interestingly, for a highly oriented defect-free GaS, the calculated spectra indicate a transmittance of 99.9% for the monolayer, 94.75% for approximately 10 layers, and 82.3% for approximately 20 nm (i.e., 20 layers), approaching the bulk 80% transmittance for a thickness higher than 20 nm. The measured optical transmittance line shape for the 30–40 nm GaS is in good agreement with the simulations performed for GaS layers with thickness in the range 10–50 nm. These spectra are characterized by a pronounced dip at ≈3.9 eV consistent with the interband critical point GS2 in the dielectric function as reported by Schlüter et al. (1976) and Isik et al. (2013). In the low-energy range, the lower measured transmittance as compared with simulation can be associated with phenomena not considered in the model such as surface roughness, scattering, as well as inhomogeneities in the sample. In the case of the ≈5-µm layer, the main difference between the measured and simulated spectra is in the onset and slope of the transmittance around the energy bandgap and the damped interference system. In this thick case, these differences can likely be attributed to polycrystallinity and defects or doping of the sample. This is supported by comparing the optical transmittance obtained by the model and that measured in a single crystal by Nakamura et al. (2021), in which the spectra also show a sharp step around the energy bandgap. These results provide evidence about the high density of defects introduced in the layers by the mechanical exfoliation process, which are also potentially the radiative traps causing the PL in Figure 3C for the thick GaS layers.

Furthermore, GaS has been demonstrated to be exploitable in blue-UV photodetectors (Chen et al., 2019; Lu et al., 2020). A critical parameter to be considered in the photodetector design is the resistivity of the active layer under light irradiation. Therefore, the photoresistivity of layered GaS with thickness ranging from ≈40 to 1900 nm transferred onto glass was also investigated. The calculated resistivity as a function of the GaS thickness with and without light irradiation is shown in Figure 4B.

In the dark state, GaS shows a typical resistivity of ≈10^7^ Ω·cm independently of the thickness. This value is consistent with those reported by other authors (Manfredotti et al., 1976; Mancini et al., 1983; Micocci et al., 1990; Szałajko and Nowak, 2007). When illuminated, the GaS resistivity decreases for all the investigated thicknesses, consistently with an increase of the light absorption depth and generation of photocurrent in the GaS layers. Furthermore, the resistivity is lower when samples are illuminated by UV light than visible light. The lower resistivity values obtained under UV indicates a larger amount of free carriers generated due to the fact that all the excitation photons have an energy (405 nm/3.05 eV) above the GaS bandgap, making the electron–hole pair generation more efficient than in the case of the visible light source, for which part of the emission spectrum contains photons below the GaS bandgap unable to generate photocarriers.

### Thickness Dependence of the Work Function

**FIGURE 5:**
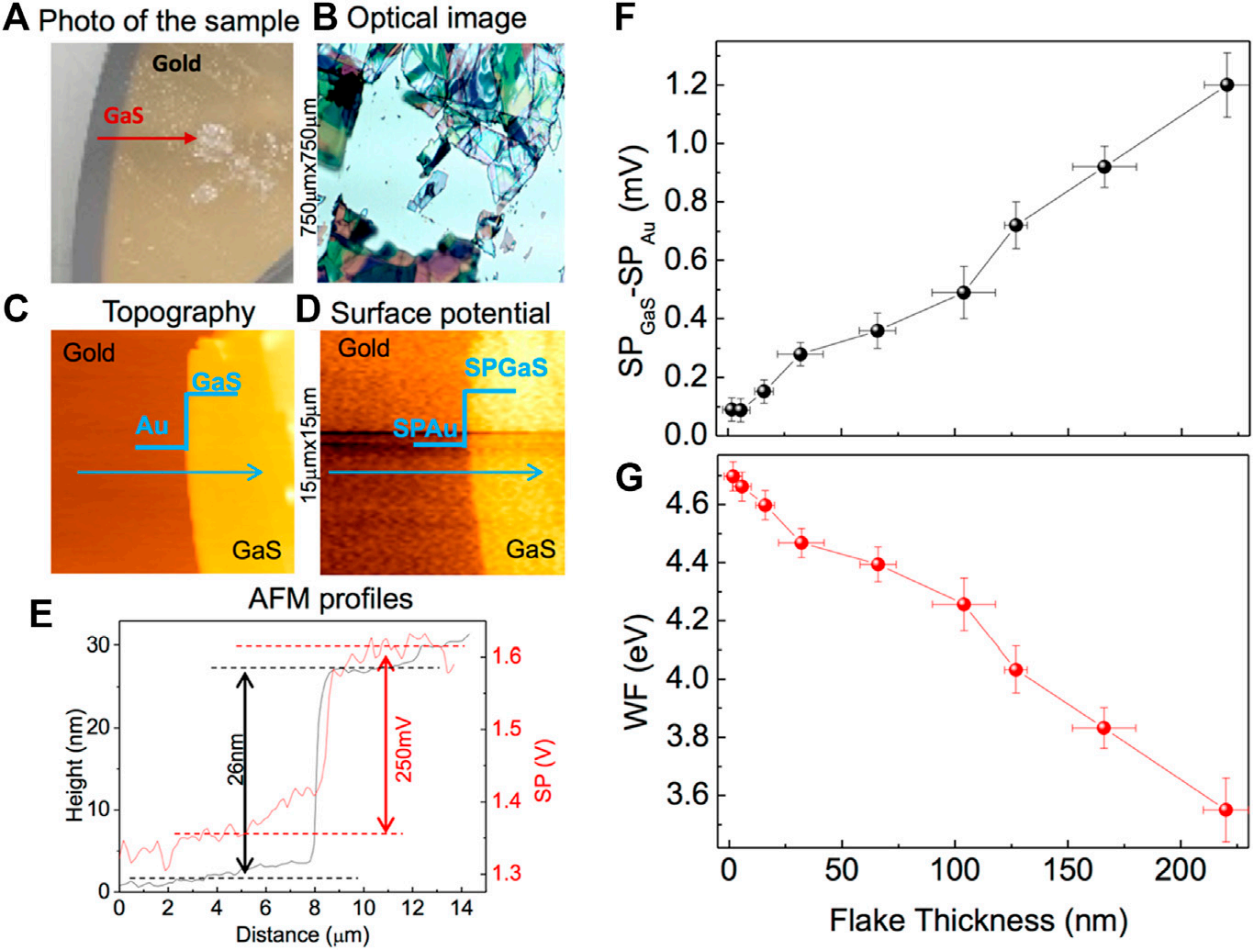
**(A)** Picture of GaS flakes on gold; **(B)** optical image of a 750 μm × 750 μm area of GaS flakes **(C,D)** examples of 15 μm × 15 µm topographic and SP maps for a 26-nm-thick GaS flake. **(E)** Representative topographical (black line) and SP (red line) profiles for a GaS flake with 26-nm thickness against gold. **(F)** Thickness dependence of the difference in surface potential (SP) between gold and GaS. **(G)** Thickness dependence of the GaS work function (WF).

Figure 5 illustrates the use of KPFM to determine the WF of GaS layers with different thickness, as it appears from the optical image (Figure 5B) of the sample, where the different colors correspond to flakes of different thickness, which have been mapped by atomic force topographies. The exfoliated GaS was deposited on a gold substrate, as shown in Figure 5A. A typical topography and corresponding surface potential SP map, 15 × 15 μm, are shown in Figures 5C,D, respectively. The difference between the SP of GaS flake, SP_GaS_, and the SP of the gold, SP_Au_, quantifies the difference in their Fermi levels according to the following relation:

$$\Delta SP=SP_{\mathrm{GaS}}-SP_{\mathrm{Au}}=WF_{\mathrm{tip}}\mathrm{-W}F_{\mathrm{GaS}}\mathrm{-W}F_{\mathrm{tip}}\mathrm{+W}F_{\mathrm{Au}}=WF_{\mathrm{Au}}\mathrm{-W}F_{\mathrm{Gas}}$$ (1)

where WF_tip_ is the tip work function, WF_Au_ is the gold work function, and WF_GaS_ is the work function of the GaS flake.

Figure 5E shows representative topographic and SP profiles obtained for a GaS flake with 26-nm thickness against gold. Specifically, the SP of the GaS flake is higher than the Au SP, i.e., SP_GaS_ > SP_Au_, corresponding to a WF of the GaS flake 250 meV lower than that of the gold reference, which we previously calibrated to be 4.75 eV (Giangregorio et al., 2015).

By plotting the difference between the surface potential of the GaS flakes with a known thickness, SP_GaS_, and the surface potential of gold, SP_Au_, and the corresponding WF_GaS_, as shown in Figures 5F,G, we found that GaS layers always have a SP higher than Au independently of their thickness. Moreover, the WF_GaS_ increases with the decrease in the GaS thickness from 3.55 ± 0.10 eV for GaS bulk to 4.70 ± 0.05 eV for GaS monolayer.

In order to explain this trend of the WF, we consider that 1) going from bulk to monolayer, the WF becomes more and more sensitive to the chemical and physical conditions at its surface, and 2) it depends on crystalline orientations, surface contamination, and surface roughness, which induce stress fluctuations affecting the Fermi level as well as the electrostatic potential in the vicinity of surface. It is worth pointing out that adsorption of atoms or molecules on GaS changes the surface dipole layer and hence the WF; e.g., electronegative adsorbates (e.g., O, C, and S) increase the WF. Oxygen adsorption involves localized orbital overlap and charge transfer between the adsorbate and surface atoms. The GaS bulk WF value is small compared with the oxygen ionization energy (13.618 eV), causing electron transfer from the GaS layer. Consequently, the WF increases as the negative pole of the adsorded oxygen molecule points toward the vacuum, so the surface space charge or surface dipole presents an electrostatic field that causes an increase in the WF. The effect of adsorbates becomes more important with the decrease in the number of layers down to monolayer. The presence of those C and O adsorbates was confirmed by x-ray photoelectron spectroscopy (XPS).

**FIGURE 6:**
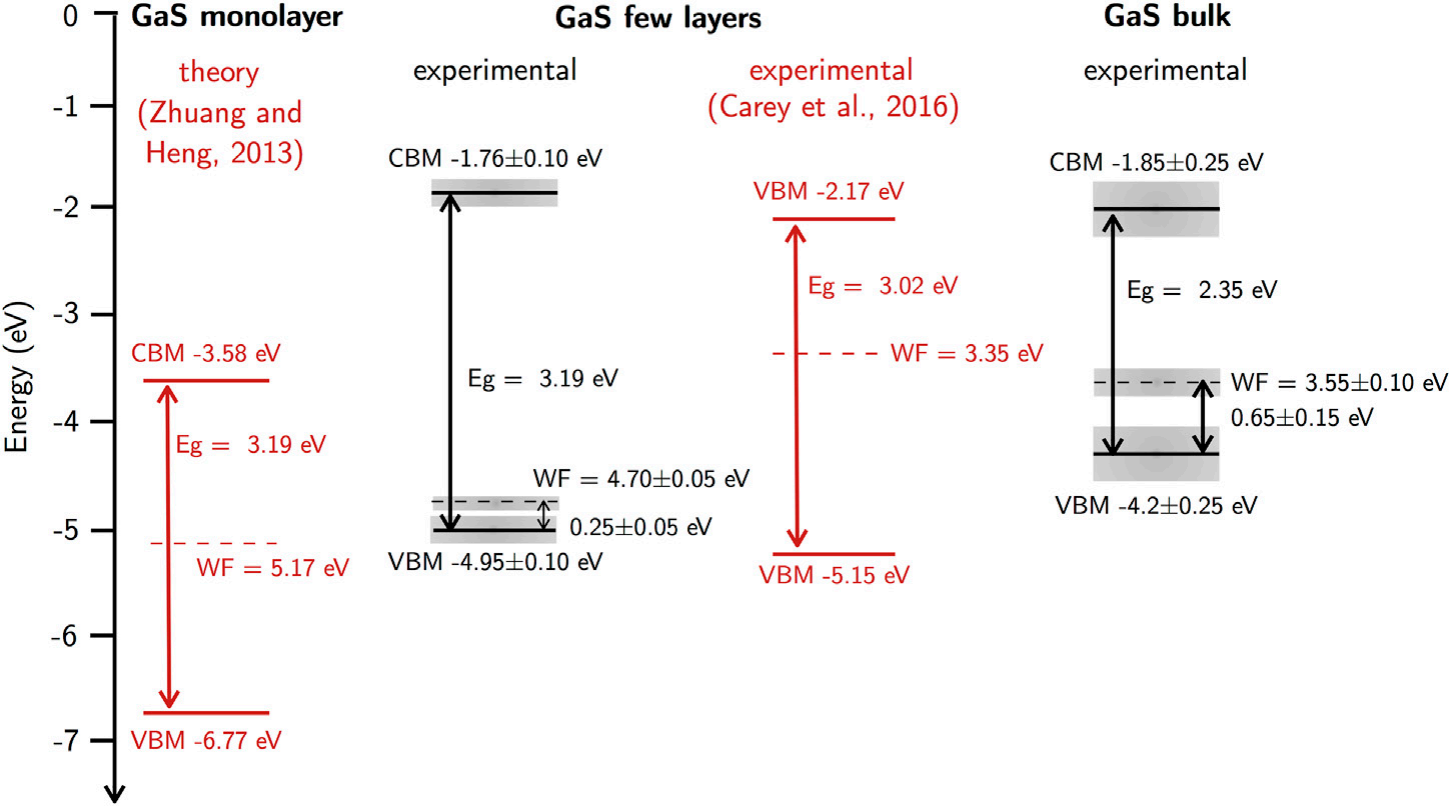
Profiling of the valence band maximum (VBM), conduction band minimum (CBM), and Fermi level with respect to the vacuum level for GaS monolayer, few layers, and bulk considering theoretical (Zhuang and Hennig, 2013; Carey et al., 2017) and experimental values.

These measurements of WF are useful to profile the bands and Fermi level variations as a function of GaS thickness. For monolayer GaS, we consider as reference the values calculated by Zhuang and Hennig (2013) of the bandgap E_g_ = 3.19 eV, of the valence band maximum VBM = −6.77 eV and of the conduction band minimum CBM = −3.58 eV, as shown in Figure 6. In profiling the bands, the WF values were complemented with experimental values of the difference of the VBM with respect to Fermi level derived from the XPS valence band analysis. In the case of the few-layer GaS, we profiled the measured values of the WF and E_F_ of 4.70 ± 0.05 eV and of the VBM position with respect to Fermi level of 0.25 ± 0.05 eV. Assuming an energy bandgap similar to that of GaS monolayer, the VBM and CBM were set at −4.95 ± 0.10 and −1.76 ± 0.10 eV, respectively. For comparison, the values of VBM, CBM, and E_F_ reported by Carey et al. (2017) for few-layer GaS are also shown. In the case of bulk GaS, the measured WF = 3.55 ± 0.10 eV, the difference of 0.65 ± 0.15 eV for the VBM with respect to E_F_, and the energy bandgap of 2.35 eV led to VBM and CBM at −4.2 ± 0.25 and −1.85 ± 0.25 eV, respectively. From these bands and Fermi level profiling, it can be inferred that the analyzed GaS samples are p-type semiconductors. This is consistent with other studies on GaS crystals grown by the Bridgman method (Lieth et al., 1969). As an example, for GaS monolayer, it has been reported that it becomes p-type under the gallium-poor and sulfur-rich conditions (Chen et al., 2015). The adsorbates mentioned above as well as the interface traps mentioned in Figure 3C could also contribute to the p-type doping of exfoliated GaS layers. The detailed identification of those radiative traps and p-type doping defects is in progress.

## Conclusion

GaS layers of different thickness have been exfoliated and transferred to glass substrates. Different properties, such as structural properties from Raman spectra, PL, optical transmittance, resistivity, and WF have been investigated as a function of the number of layers. The Raman spectra measured in layers with thickness ranging from the monolayer to 120 nm show no significant variation in the peak position and broadening, whereas their intensity is proportional to sample polarizability and, hence, increases with thickness. A model based on a planar stack of layers is able to reproduce the line shape of the optical transmittance spectra for few layers and micron-thick GaS layers. Phenomena of surface roughness, inhomogeneities, defects, or unintentional doping clearly decrease the transmittance. GaS dark resistivity is in the range of ≈10^7^ Ω·cm, independently of the thickness. Under visible and UV illumination, the resistivity decreases, and a pronounced dependence on GaS thickness is found. Finally, the analysis of the WF, using Kelvin probe force microscopy, shows an increase in the WF going from Bulk GaS down to monolayer. Accordingly, GaS bands have been profiled as a function of thickness. Although the study on this new 2D material is in progress, those trends can be useful to design optoelectronic devices based on GaS.

## Data Availability

The original contributions presented in the study are included in the article/Supplementary Material, further inquiries can be directed to the corresponding author.
